# Novel Framework for Assessing Epidemiologic Effects of Influenza Epidemics and Pandemics 

**DOI:** 10.3201/eid1901.120124

**Published:** 2013-01

**Authors:** Carrie Reed, Matthew Biggerstaff, Lyn Finelli, Lisa M. Koonin, Denise Beauvais, Amra Uzicanin, Andrew Plummer, Joe Bresee, Stephen C. Redd, Daniel B. Jernigan

**Affiliations:** Author affiliation: Centers for Disease Control and Prevention, Atlanta, Georgia, USA

**Keywords:** influenza, pandemic, preparedness, viruses, epidemiology, epidemic

## Abstract

The effects of influenza on a population are attributable to the clinical severity of illness and the number of persons infected, which can vary greatly between seasons or pandemics. To create a systematic framework for assessing the public health effects of an emerging pandemic, we reviewed data from past influenza seasons and pandemics to characterize severity and transmissibility (based on ranges of these measures in the United States) and outlined a formal assessment of the potential effects of a novel virus. The assessment was divided into 2 periods. Because early in a pandemic, measurement of severity and transmissibility is uncertain, we used a broad dichotomous scale in the initial assessment to divide the range of historic values. In the refined assessment, as more data became available, we categorized those values more precisely. By organizing and prioritizing data collection, this approach may inform an evidence-based assessment of pandemic effects and guide decision making.

## Abstract 2

Organizing and prioritizing data collection may lead to informed assessment and guide decision making.

Pandemic influenza results from the emergence of a new influenza A virus to which the population possesses little or no immunity (*1*). Past pandemic influenza viruses have spread rapidly worldwide, affecting persons of all ages and causing substantial illness and death. Influenza can result in a wide spectrum of clinical outcomes in infected persons, including asymptomatic infection, medically and non–medically attended respiratory illness, hospitalization, or death. The likelihood of these outcomes is variable and depends on many factors, including the age of the patient, the presence of underlying medical conditions, and characteristics of the virus itself (*2*).

The overall number of illnesses and deaths from influenza in the population may be primarily attributable to a combination of both the clinical severity of illness in infected persons and the transmissibility of the infection in the population. Figure 1 shows the increasing expected number of deaths in the US population as both the cumulative incidence of influenza in the population and the case-fatality ratio (CFR) increase.

**Figure 1:**
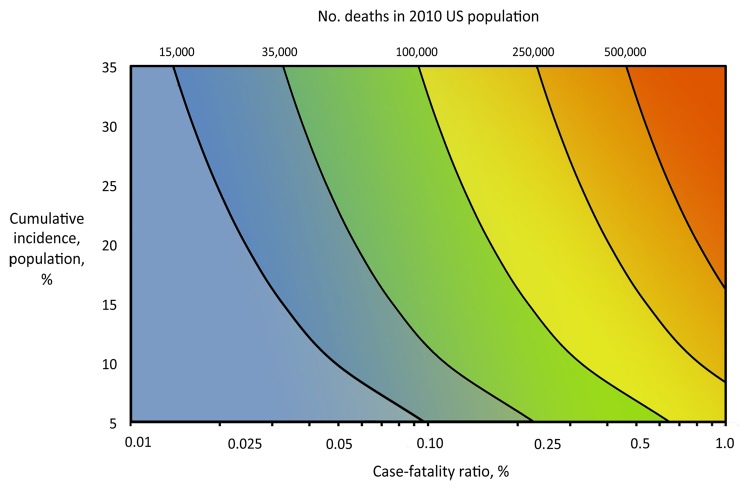
Estimates of influenza deaths in the 2010 United States population (308,745,538 persons) across varying values of case-fatality ratio and the cumulative incidence of infection in the population. Selected estimated numbers of deaths are indicated with a black line, across each relevant combination of case-fatality ratio and cumulative incidence. In addition, the background color transitions from blue to yellow to red as the estimated absolute number of deaths increases.

Because the risk for severe outcomes and differences in the rates of transmission of the virus can vary, the effects on the population observed during pandemics have ranged from those similar to severe seasonal influenza epidemics to those experienced during the 1918 influenza pandemic. Depending on the overall population effects, a pandemic could overwhelm the capacities of public health and health care systems or result in societal disruption because of school or workplace absenteeism, which could affect critical infrastructure (*1*,*3*).

Historically, assessment of influenza pandemic effects has been characterized by using an estimate of the overall CFR (*4*). Although this approach provided guidance for planning and projections of the expected number of deaths from pandemic influenza in the population, using that ratio alone presents several challenges. First, deaths from influenza may occur weeks after illness begins and can also be subject to reporting bias, delaying the ability of public health and government leaders to quickly issue recommendations for evidence-based public health interventions if they lack an accurate estimate of CFR. Second, a single overall CFR does not fully account for the varying effects a seasonal epidemic or pandemic could have on vulnerable population subgroups, which could include children or the elderly, those with chronic conditions, or certain racial and ethnic minorities. Finally, CFR does not address other societal effects, such as absenteeism or the demand on health care services from excess outpatient visits and hospitalizations, that could result from increased transmission. Because of these limitations, relying on CFR as a single measure of the effects on a population may make an assessment difficult if such data are not yet available early in a pandemic or misleading if the available data are not well characterized and the biases are not well understood.

The ability to synthesize epidemiologic data collected early during a pandemic to characterize its anticipated public health effects is of vital importance to public health officials in the United States and worldwide. Here we provide a conceptual framework with which to characterize the expected effects of a pandemic in the context of past experience with influenza epidemics and pandemics in the United States. We examined published data from past influenza seasons and pandemics to determine the range of effects of influenza in the United States. The framework provides a basic structure by which to synthesize epidemiologic data and on which preparedness plans can be developed to guide and communicate the pandemic influenza response.

## Methods

We developed the assessment framework using a 4-step process. The steps included were the following: 1) identify and evaluate available measures of influenza transmissibility and severity, 2) create a standard scale for selected measures, 3) summarize and scale available measures, and 4) provide historical context. 

### Step 1: Identify and Evaluate Measures of Transmissibility and Severity

We first identified epidemiologic measures that may be indicators of either the transmissibility of a novel influenza virus or the clinical severity in infected persons. The identification of relevant measures within these categories was based on an extensive review of historical seasonal and pandemic influenza literature, including published articles and reports of surveillance data collected from the 1918 pandemic forward. Three criteria were used to evaluate the identified measures: 1) the availability and quality of data related to the measures during the early stages of past influenza pandemics and seasonal influenza epidemics; 2) the presence of enough variation in the measure to produce a biologically plausible and measurable scale; and 3) the epidemiologic strengths and limitations of the measure (Technical Appendix).

### Step 2: Scaling Measures of Transmissibility and Severity

From the list of measures identified in step 1, we abstracted data from the literature review on the measures as reported during previous influenza seasons and pandemics. To create a comparable scale across the various measures of transmissibility and clinical severity, we first identified the range of values that had been observed historically for each measure. The data for each measure were then categorized into a uniform scale that was consistent across indicators of transmissibility and across indicators of clinical severity.

Because the availability and quality of epidemiologic information will increase throughout the course of a pandemic, we divided the assessment process into 2 assessment frameworks: 1) an “initial assessment” when data are sparse or very uncertain, and 2) a “refined assessment” when data are more available and more certain. A uniform scale of the transmissibility and clinical severity indicators was developed for each framework. When transmission of a novel influenza virus is identified, early epidemiologic measures provide a broad initial assessment, albeit with a high level of uncertainty, and were categorized by using a broad dichotomous scale. The assessment framework would become more refined as additional epidemiologic and clinical information are gathered and the biases in the earliest measures are better characterized. During this period, a similar general framework would incorporate a finer scale, allowing for more discrete separation of seasonal epidemics and pandemics.

### Step 3: Summarize and Score Available Measures

**Figure 2:**
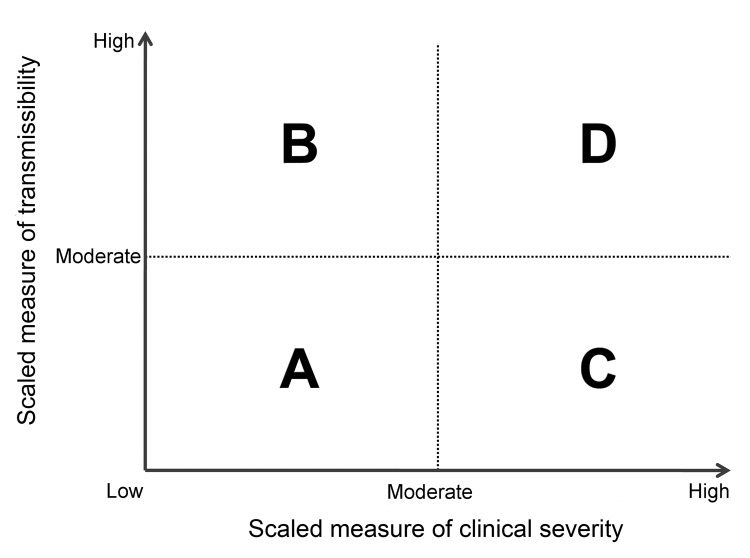
Framework for the initial assessment of the effects of an influenza pandemic.

**Figure 3:**
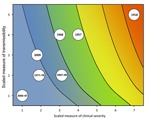
Framework for the refined assessment of the effects of an influenza pandemic, with scaled examples of past pandemics and past influenza seasons. Color scheme included to represent corresponding estimates of influenza deaths in the 2010 US population as shown in Figure 1.

**Figure 4:**
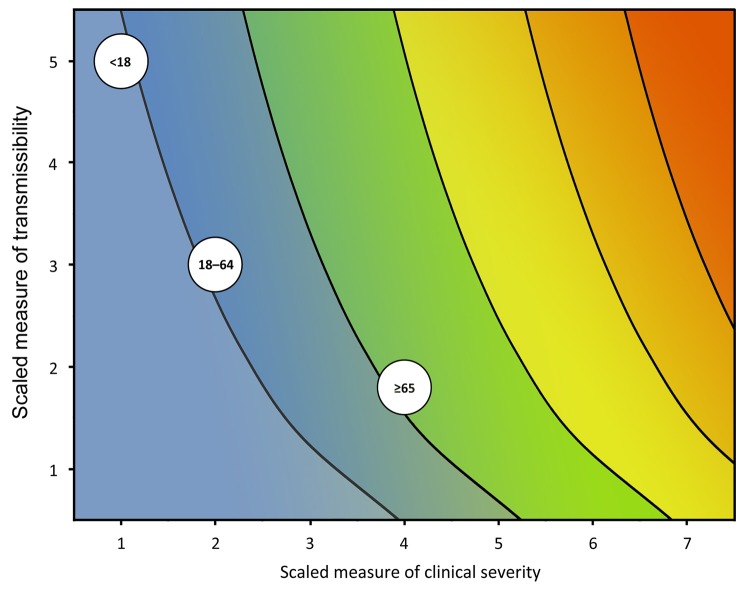
Framework for the refined assessment of the effects of an influenza pandemic, stratified by age group with scaled examples from the 2009 pandemic. Color scheme included to represent corresponding estimates of influenza deaths in the 2010 US population as shown in Figure 1.

During the initial assessment, a combination of the dichotomous scale for indicators of transmissibility and the dichotomous scale for indicators of severity results in a framework with 4 profiles (A, B, C, D) (Figure 2). An initial assessment can be made as soon as data on some measures become available and would continue to be reviewed and revised as the data warrant. As early data become available, issues of data quality are also essential to consider; we include a list of such considerations in the Technical Appendix. Once more robust data are available, the assessment could transition to the more detailed scale of the refined assessment framework, with scaled values of severity and transmissibility plotted along an x-axis and y-axis, respectively (Figure 3). Because the effects of an influenza pandemic may vary between age groups, the refined assessment could also be conducted with age-stratified data on indicators of transmissibility and clinical severity and then plotted by using the same scale and framework (Figure 4).

### Step 4: Provide Historical Context

For the refined assessment, we scaled and plotted data from obtained from our literature riew for 4 pandemics (2009, 1968, 1957, 1918) and 3 nonpandemic influenza seasons that ranged in transmissibility and severity (1978–79, 2006–07, and 2007–08) (Technical Appendix). When multiple measures for transmissibility or severity were present, we used the median score across all available measures. Age-stratified data from the 2009 influenza A (H1N1) pandemic were also similarly scaled and plotted by using the age categories <18 years, 18–64 years, and >65 years.

## Results

### Initial Assessment

Early in a pandemic, the spread of a novel virus is likely to be restricted to a particular geographic area, mostly in focal clusters of infections, and epidemiologic data are limited. To reflect the uncertainty in early data, we divided each measure of transmissibility and severity for the initial assessment framework into a dichotomous scale corresponding to the low-moderate and moderate-high ends of the range of values from the literature review. Scaled values for the initial assessment are shown in Table 1.

**Table 1:** Scaled measures of transmissibility and clinical severity for the initial assessment of pandemic influenza effects

Parameter no. and description	Scale	
	Low-moderate	Moderate-high
Transmissibility		
1. Secondary attack rate, household, %	<20	>20
2. Attack rate, school or university, %	<30	>30
3. Attack rate, workplace or community, %	<20	>20
4. R_0_: basic reproductive no.	1.0–1.7	>1.8
5. Underlying population immunity	Some underlying population immunity present	No underlying population immunity present
6. Emergency department or other outpatient visits for influenza-like illness, %	<10	≥10
7. Virologic characterization	Genetic markers for transmissibility absent	Genetic markers for transmissibility present
8. Animal models—transmission studies	Less efficient or similar to seasonal influenza	More efficient than seasonal influenza
Clinical severity		
1. Upper boundary of case-fatality ratio, %	<1	>1
2. Upper boundary of case-hospitalization ratio, %	<10	>10
3. Ratio, deaths: hospitalizations, %	<10	>10
4. Virologic characterization	Genetic markers for virulence absent	Genetic markers for virulence present
5. Animal models	Less virulent or similar to seasonal influenza	More virulent than seasonal influenza

We recognized that early measures are likely to have substantial biases. Early measures of the transmissibility of the virus are likely to come from larger recognized outbreaks, which may lead to higher estimates than would eventually occur in the whole population. Likewise, early indicators of severity may be overestimated if severe illnesses are more likely to be recognized, as was seen worldwide early in the 2009 influenza A (H1N1) pandemic (*5*,*6*). For example, reports to the Centers for Disease Control and Prevention (Atlanta, GA, USA) of confirmed cases in the first few weeks of the 2009 pandemic indicated a crude CFR of 0.3% (*7*), ≈10-fold higher than it was estimated to be following adjustment for underdetection (*5*,*8*). To account for this bias in early measurements, we set the midpoint of the CFR in the initial assessment 10× higher than the midpoint in the refined assessment.

Early measures of transmissibility were then scaled along a y-axis, and early measures of clinical severity were scaled along an x-axis. From the combination of these 2 dichotomous scales, the initial framework results in 4 quadrants (Figure 2). In quadrant A, for example, available indicators appear similar to the range seen in annual seasonal epidemics. For quadrant B, although clinical severity is in the range of that seen in seasonal epidemics, the transmissibility is greater and thus overall rates of severe outcomes may be greater. Conversely, in quadrant C, transmissibility is similar to that of seasonal epidemics, but severity is expected to be higher, again leading to increased expected rates of severe outcomes, but for a different reason. Finally, in quadrant D, both indicators are greater than expected during annual seasonal epidemics. Consequently, recommended guidance and interventions during the pandemic response may be different between the quadrants.

### Refined Assessment

Although the assessment would be updated routinely as new data become available, an increase in the amount and quality of data will allow results to be presented in a more precise, refined assessment. For this framework, the range for each measure of transmissibility was divided into a 5-point scale while the range for each measure of clinical severity, which covered a broader range of values, was divided along a 7-point scale. To illustrate this assessment framework, we selected 5 measures of transmissibility and 3 measures of severity to scale on the basis of information obtained in our literature review. Detailed discussions of the measures and their strengths and limitations are in the Technical Appendix. Table 2 displays the ordinal scales for the measures of transmissibility and clinical severity that we developed for the refined assessment. For example, a cumulative symptomatic attack rate of 12% would be classified as a 2 on the scale, whereas a cumulative symptomatic attack rate of 28% would be a 5 on the scale. Likewise, a CFR of 0.01% would be a 1 on the clinical severity scale, whereas a CFR of 1.2% would be a 7. Each measure followed this approach with a scale of 1, representing the lowest observed values for that parameter, with values increasing as the scale increases.

**Table 2:** Scaled measures of transmissibility and clinical severity for the refined assessment of pandemic influenza effects

Parameter no. and description	Scale						
	1	2	3	4	5	6	7
Transmissibility							
1. Symptomatic attack rate, community, %	<10	11–15	16–20	21–24	>25		
2. Symptomatic attack rate, school, %	<20	21–25	26–30	31–35	>36		
3. Symptomatic attack rate, workplace, %	<10	11–15	16–20	21–24	>25		
4. Household secondary attack rate, symptomatic, %	<5	6–10	11–15	16–20	>21		
5. R_0_: basic reproductive no.	<1.1	1.2–1.3	1.4–1.5	1.6–1.7	>1.8		
6. Peak % outpatient visits for influenza-like illness	1–3	4–6	7–9	10–12	>13		
Clinical severity							
1. Case-fatality ratio, %	<0.02	0.02–0.05	0.05–0.1	0.1–0.25	0.25–0.5	0.5–1	>1
2. Case-hospitalization ratio, %	<0.5	0.5–0.8	0.8–1.5	1.5–3	3–5	5–7	>7
3. Ratio, deaths: hospitalization, %	<3	4–6	7–9	10–12	13–15	16–18	>18

Using available measures of transmissibility and clinical severity and the scale in Table 2, we plotted the coordinates for several sample years on the refined assessment framework. For example, using the 2009 pandemic (Table 3), available measures of clinical severity included the symptomatic CFR, the symptomatic case-hospitalization ratio, and the ratio of deaths to hospitalizations (*5*,*8*). Each of these measurements was a 2 on the ordinal scale of clinical severity. Available measures of transmissibility from 2009 included a household secondary attack rate (*9*–*11*), an estimated population clinical attack rate (*12*), an estimated R_0_ (*13*), and a peak percent of visits for influenza-like illness from national surveillance (*14*). Each of these measurements was a 3 on the scale of transmissibility. This is illustrated at the coordinate (*2*,*3*) in Figure 3. We likewise characterized data abstracted from past pandemics and selected previous seasons and also plotted them as shown in Figure 3. Further details are included in the Technical Appendix.

**Table 3:** Indicators of severity and transmissibility from the 2009 influenza (H1N1) pandemic and the corresponding assessment scale

Parameter	Value	Score
Clinical severity		
Symptomatic case-fatality ratio, %	0.02	2
Symptomatic case-hospitalization ratio, %	0.05	2
Ratio, deaths: hospitalization, %	4.7	2
Overall		2
Transmissibility		
Household secondary attack rate, symptomatic, %	13	3
Symptomatic attack rate, community, %	20	3
Peak % visits for influenza-like illness	7	3
R_0_: basic reproductive no.	1.4	3
Overall		3

In addition, we abstracted and scaled data from the 2009 pandemic by age group. These values were plotted in Figure 4, with the dashed box representing the overall assessment of the 2009 pandemic. As shown, the available data indicated that persons <18 years of age had a high incidence of infection during the pandemic (an overall symptomatic attack rate of 26% [*12*], 5 on the transmissibility scale), but relatively few in that age group who became ill died (a CFR of 0.005% [*5*,*8*], 1 on the clinical severity scale). Those >65 years of age, however, had little illness (an overall symptomatic attack rate of 15% [*12*], 2 on the transmission scale), but more of those who became ill died (a CFR of 0.18% [*5*,*8*], 4 on the clinical severity scale). Persons 18–64 years of age had values that were similar to those of the overall assessment.

## Discussion

A new framework to assess pandemic effects was developed to systematically assess the potential population effects of an influenza pandemic by characterizing data on both transmissibility and clinical severity and providing historical context from past pandemics and influenza seasons. We divided the framework into 2 periods. In the initial assessment, during the early stages of a pandemic, few epidemiologic data may be available and early indicators can be variable. These indications were thus categorized by using a broad dichotomous scale. In the refined assessment, as increased data become available later in a pandemic, the ranges of transmissibility and severity measures were more finely categorized.

Rather than rely only on a single measure, such as the CFR, to assess the potential effects of a pandemic, which may be misleading if those data are unavailable or not representative early in the pandemic, we incorporated several epidemiologic measures into the framework, although the CFR remains a valuable measure of clinical severity. With the creation of a standard scale that includes multiple epidemiologic measures, a variety of data may be incorporated to help synthesize these different measures into an overall indicator of transmissibility and clinical severity.

The visualization of epidemiologic data in the framework provides epidemiologists, public health officials, and policy makers with an evidence-based assessment of influenza transmissibility and clinical severity in the context of previous influenza seasons and pandemics. Although the 3 selected influenza seasons are positioned in a cluster in the lower left of Figure 3, discernible differences exist between the seasons. During the 2006–07 season, subtype A/H1N1 viruses predominated (*15*), producing what has been generally regarded as a milder season in the United States; this season received the lowest score for both transmissibility and clinical severity. Conversely, during the 2007–08 season, subtype A/H3N2 viruses predominated (*16*) to produce what has been generally regarded as a more severe season. This season is positioned toward the center of the graph, which indicates greater transmissibility and clinical severity than was seen in 2006–07. The 3 modern pandemics (2009, 1968, and 1957) are clustered in the upper center of the graph, indicating that these pandemics had higher transmissibility but that overall clinical severity was either at or moderately above the level observed during some recent influenza seasons. In contrast, the 1918 pandemic was positioned at the upper right corner of the graph, indicating a very transmissible and clinically severe pandemic with extensive effects in the population.

An evidence-based assessment of pandemic effects is essential to inform decision makers early in a pandemic and enable them to develop and communicate preventive recommendations to reduce illness and death. The context provided by the assessment of transmissibility and severity can inform the selection of pharmacologic and nonpharmacologic interventions that may be appropriate to mitigate the anticipated effects of a pandemic. For example, although the early initial assessment was categorized into only 4 quadrants, this broad early assessment can help organize available information to facilitate early decision-making that may need to be initiated when data are still limited. When clinical severity is high (quadrants C/D), measures may be initiated to provide early treatment to all who are ill and to reduce spread to limit severe disease outcomes and demand on health systems. If clinical severity appears to be similar to seasonal epidemics, but incidence is high (quadrant B), measures may be taken to reduce transmission and prepare for the possibility of disruption in schools and workplaces due to absenteeism. As more data are collected, the assessment transitions into a more detailed refined assessment, and a better characterization of the risks of transmissibility and clinical severity. Subsequently, recommendations and communications may be refined to better reflect the potential effects of the evolving pandemic. Work is ongoing at the Centers for Disease Control and Prevention to use the assessment framework to select different combinations of transmissibility and clinical severity and develop prepandemic guidance on the basis of the potential effects in the population.

Although this framework provided an assessment of the potential population effects from an influenza pandemic, it should not be used in isolation of other epidemiologic data. As this study illustrated, the assessment may be stratified to incorporate data on transmissibility and severity by age group or other risk factors to assess how the expected effects might vary in and across these groups. In addition, decision makers should consider the potential effects in relation to the time at which the pandemic emerges and the particular course of the epidemic in an area (i.e., early vs. approaching peak activity). For example, although the United States experienced a peak of pandemic activity in the late spring of 2009, for most of the country that wave ultimately accounted for only ≈5%–8% of the total estimated burden of influenza during the first year of the pandemic (*5*,*12*). Decision makers should also consider additional factors that are relevant to their individual communities, regions, and states when formulating guidance for interventions based on the epidemiologic impact assessment. These considerations include factors such as access to adequate health care and public health interventions among the affected population, the demographic make-up, the presence of vulnerable populations, or the population density.

Our assessment is subject to some limitations. We conducted a literature review of published data on measures of transmissibility and clinical severity from past influenza seasons and pandemics. Some data were sparse or contradictory, making it difficult to fully understand the variability within measures and the comparability between measures. However, building the framework around a standard scale provides flexibility to refine how measures are categorized as additional data become available and allows for other measures to also be incorporated into the scale. This lack of data underscores the need for ongoing study of the epidemiology of annual epidemics of influenza to improve our ability to accurately characterize the variability in the transmissibility and severity of influenza. An increased understanding of the effects of seasonal influenza will help the public health community prepare for the potential effects of a novel influenza virus.

In addition, there will be biases and limitations in the measurement or availability of epidemiologic data to incorporate in the framework. The Technical Appendix describes an evaluation of several epidemiologic measures and available data sources. We attempted to account for some of the known biases by adjusting the scales used in the initial assessment on the basis of the most recent experience of the 2009 pandemic. However, changes in care-seeking behavior or testing practices may require readjusting the scale to more accurately reflect future trends. It is also possible that severity could be underestimated initially because of the delay from illness to death, which we did not directly account for (*17*). In the case of influenza, however, this underestimation may have less bearing than the substantial underrecognition of community transmission (*6*).

Continued refinement of the methods by which we collect and analyze data annually on influenza will improve our ability to have accurate and reliable data during a pandemic. A key challenge in assessing the effects of an influenza pandemic is that many cases of influenza are mild, even in the most severe pandemics, and not all persons will seek medical care or be tested for influenza. This leads to an underestimation of the incidence by missing persons who do not seek medical care and biases estimates of severity by disproportionately detecting more severe cases. Developing novel methods to better characterize the community effects of influenza will be vital to define a more accurate case denominator. In addition, strengthening systematic surveillance methods and better characterizing existing systems will also help address some of the biases in the detection of influenza and the estimation of key epidemiologic parameters.

Although we used data from the United States, the framework provides a basic structure to synthesize epidemiologic data that may be useful in other settings as well. The measures used to characterize epidemics and pandemics of influenza have both strengths and limitations; thus, we developed a the framework that is flexible and can be adapted over time to incorporate or refine measures as more data become available or better characterized. Further evaluation of the framework will be needed to determine whether it will be used as a formal policy for pandemic planning and response. This standardized approach informs the assessment of pandemic impact by organizing available epidemiologic information using a set of key parameters to prioritize data collection and facilitate decision making.

Technical Appendix Description of process used to evaluate measures of influenza transmissibility and severity characterized historically in the literature.  Parts A and B review measures that could be used to characterize novel influenza viruses and pandemics and include a detailed discussion of their strengths and limitations.  Part C outlines several data quality issues that should be considered in the inclusion of data in the assessment framework.  Finally, Part D provides additional detail on the data abstracted from the literature on past pandemics and selected seasons that were used to scale examples provided in the manuscript.
