# Supplementary material for: Novel Framework for Assessing Epidemiologic Effects of Influenza Epidemics and Pandemics
Source: Emerg Infect Dis. 2013 Jan;19(1):85–91. doi: 10.3201/eid1901.120124 (PMC3557974; doi:10.3201/eid1901.120124)
Supplement: Technical Appendix — Description of process used to evaluate measures of influenza transmissibility and severity characterized historically in the literature. Parts A and B review measures that could be used to characterize novel influenza viruses and pandemics and include a detailed discussion of their strengths and limitations. Part C outlines several data quality issues that should be considered in the inclusion of data in the assessment framework. Finally, Part D provides additional detail on the data abstracted from the literature on past pandemics and selected seasons that were used to scale examples provided in the manuscript. [file 12-0124-Techapp-s1.pdf]

Article DOI: http://dx.doi.org/10.3201/eid1901.120124

Novel Framework for Assessing Epidemiologic Effects of Influenza Epidemics and Pandemics

Technical Appendix

To develop the framework presented in the manuscript we evaluated several measures of influenza transmissibility and severity that have been characterized historically in the literature. In parts A and B, we provide a review of those measures that could be used to characterize novel influenza viruses and pandemics, including a detailed discussion of their strengths and limitations. Some measures did not have sufficient historical data, and were not able to be included in the assessment framework. Such measures may be incorporated into the framework in the future as they become better characterized. In Part C, we outline several data quality issues that should be considered in the inclusion of data in the assessment framework. Finally, in Part D we provide additional detail on the data abstracted from the literature on past pandemics and selected seasons that were used to scale examples provided in the manuscript.

Contents

A. Evaluation of measures of transmissibility: Description of parameter sources, strengths, and limitations ... 3
1. R0 and serial interval ... 3
2. Estimated attack rate (community, household, school, workplace) ... 3
3. Medically-attended outpatient ILI visits ... 4
4. Underlying population immunity ... 4
5. Genetic markers of transmissibility ... 5
6. Animal transmission experiments ... 5
7. School/workplace absenteeism, including healthcare workers (HCW) ... 5

B. Evaluation of measures of clinical severity: Description of parameter sources, strengths, and limitations ... 6
1. Case-fatality and case-hospitalization ratios ... 6
2. Ratio of deaths to hospitalizations ... 6
3. Genetic markers of virulence ... 7
4. Animal immunopathologic experiments ... 7

5. Percent of ED visits that resulted in hospitalization 7
6. Percent of hospitalizations admitted to ICU 7
7. Rate of hospitalization... 8
8. Excess deaths... 8
C. Data quality evaluation 9
D. Data on measures of transmission and severity used to scale examples of past seasons and pandemics 10
E. References:... 14

A. *Evaluation of measures of transmissibility: Description of parameter sources, strengths, and limitations*

1. R0 and serial interval

R_0 (the basic reproductive number) is defined as the average number of secondary cases per typical case in an otherwise susceptible population. Serial interval is the time between the onset of symptoms in a case patient and the onset of symptoms in the household contacts they infect.

Strengths: These measures help to characterize the speed with which a pathogen spreads throughout the population. The magnitude of R_0 may also inform the intensity of countermeasures that may be required to halt transmission. It is possible to estimate R_0 from case incidence data alone based on the pattern of the growing incidence of cases (1), and may have reasonable precision when the incidence of cases reaches only a few hundred (2).

Limitations: There is a delay before enough cases and generations of spread have occurred to estimate these parameters reliably. Additionally, these parameters are population specific and may not be generalizable from studies that occur in different populations.

2. Estimated attack rate (community, household, school, workplace)

Strengths: Attack rate is important to calculations of morbidity and overall societal disruption due to the pandemic influenza virus. The total number of estimated cases, estimated absenteeism, and potential economic impacts rely on an accurate understanding of the number of individuals who will become ill with the new virus. Field investigations can provide a well-defined population base in which to quickly assess illness and community disruption in an affected area. Approaches such as telephone or internet surveys may allow for a rapid assessment of a relatively large population. Household studies can be a reliable source of data for estimating the secondary attack rate of the disease in households (3).

Limitations: Accurate attack rates are often difficult to estimate early on, as the selected field location must have had enough transmission to get an accurate representation of the ultimate impact of a pandemic influenza virus, and be large enough to provide reliable estimates. In local settings where significant transmission has taken place, studies may not be representative of the total population, since attack rates can vary across geographic and demographic subgroups. Confirmation of pandemic virus infection may be unlikely among all ill participants, thus case definitions that do not rely on laboratory confirmation, such as influenza-like illness, may be used. Even a carefully selected syndromic case definition will miss cases of true pandemic influenza infection, will include cases that do not have true infection.

3. Medically-attended outpatient ILI visits

Presence of an influenza-like illness (routinely defined as fever [temperature >100°F /37.8°C] with cough or sore throat) among participants is currently ascertained through a wide variety of existing surveillance networks.

Strengths: These data provide regional and national views of current influenza activity, and many surveillance systems currently function year-round. Some of these systems have substantial historic data that allow for the development of well-characterized national and regional baselines. Electronic data sources may provide a near real-time snapshot of the number of people visiting outpatient providers or ERs for influenza-like illness. Indicators from these systems are likely going to be one of the first to reflect that a pandemic virus is widespread in a community. Electronic data sources are often available, allowing accurate baselines and trends to be calculated.

Limitations: A syndromic case definition will miss cases of true pandemic influenza infection that do not cause ILI, will include cases of ILI caused by other etiologies, and will exclude asymptomatic cases, giving a limited picture of the virus' activity. In addition, estimates of medically-attended ILI can be influenced by media attention on the spread of a pandemic influenza virus. Therefore, some increase in ILI visits will be a reflection of increased care-seeking behavior where the individual might not have sought care outside of a pandemic. It may be difficult to determine this effect in the early stages of a pandemic without additional field investigations.

Finally, electronic data sources are a new and expanding source of surveillance data. Electronic health record data are not governed by a single set of standards, so each system utilized will have specific caveats and data management issues that will need to be addressed. It may be difficult to find vendors that can provide data on short notice in specific geographical areas without a pre-existing relationship. Once data are received, it may be difficult to interpret and a careful consideration of the source is warranted. Further analysis and evaluation of these data sources during annual influenza seasons will help to identify the most useful sources of data and better characterize measures and trends that would be meaningful in a pandemic situation.

4. Underlying population immunity

Strengths: If representative baseline serum samples are available, limited serologic analysis could be done in a relatively short period of time after the detection of a pandemic virus to identify whether any underlying population immunity exists to the pandemic virus.

Limitations: A dedicated, representative collection of sera with adequate geographic distribution and which captures multiple birth cohorts may not be available for rapidly determining population immunity. Additionally, serology results can be delayed by the time required to develop and conduct virus-specific serologic assays, which may require significant time and resources.

5. Genetic markers of transmissibility

Strengths: Sequencing of the viral genome and antigenic characterization will occur soon after the discovery of a pandemic influenza virus. Information such as the presence of mutations for increased propensity for transmission may be identified from the results of these analyses.

Limitations: Few mutations within the influenza virus genome cause well-defined changes in transmissibility of the virus, nor is it currently known how these mutations correlate with expected attack rate in the population. Additional research will be needed to determine how a mutation or underlying population immunity affects the transmissibility of the virus.

6. Animal transmission experiments

Strengths: Once a novel influenza virus has been isolated, experiments using ferrets or other animal models can determine if contact and respiratory transmission of the novel virus differs from the observed transmission of other seasonal, novel, or pandemic influenza viruses. This work can be accomplished relatively soon after the first detection of a novel influenza virus.

Limitations: Currently, this capability exists in only a few laboratories in the world which can carry out animal studies in appropriate conditions. Results from ferret studies may not represent transmission dynamics in humans.

7. School/workplace absenteeism, including healthcare workers (HCW)

Strengths: Significant increases in school or industrial absenteeism and overall disruption may be detected in outbreak-affected populations. If timely data are available, this may provide some proxy indicators for attack rate before the time needed to organize and conduct a more detailed investigation. Additionally, HCWs in outbreak-affected areas are likely to be one of the first groups at risk for transmission and may provide some of the first opportunities to measure transmission.

Limitations: Currently, there is limited access to historic data on absenteeism and therefore was not included in the current impact assessment. Additional data and analysis of absenteeism records and their causes will be beneficial to determine historic baselines to assess excess absenteeism during a pandemic.

B. *Evaluation of measures of clinical severity: Description of parameter sources, strengths, and limitations*

1. Case-fatality and case-hospitalization ratios

Strengths: The case-fatality ratio and the case-hospitalization ratio could be estimated from reports of early laboratory-confirmed cases to CDC. In addition, field investigations in an affected area can provide a well-defined population base in which to assess rates of morbidity and mortality in relation to the full spectrum of illness (4).

In combination with statistical expectations about the number of severe outcomes and corresponding precision in a given sample size, the occurrence or lack of severe outcomes may provide a projected range of severity and may indicate an upper bound for the estimated ratios early on.

Limitations: Early in the course of a pandemic, the availability of laboratory confirmation of infection may be unavailable to define the total number of cases, which forms the denominator of these ratios. Since not all ill people are tested, laboratory-confirmed cases will be an underestimate of total cases, and detection is likely biased to more severe cases. As a result, calculations using confirmed cases will likely overestimate the true clinical severity of infection. Novel approaches to the collection(4) and adjustment(5, 6) of data on reported cases have been proposed and may provide avenues to improve the quality of related measures early in a pandemic.

The time to hospitalization and mortality lags behind the identification of illness in the population, and investigations undertaken too quickly in a population may not adequately capture the morbidity and mortality associated with the pandemic virus.

Finally, the threshold for hospitalization can vary broadly among populations/facilities, so it may be difficult to understand how generalizable measures may be that incorporate hospitalization.

2. Ratio of deaths to hospitalizations

Strengths: If influenza testing is likely to be biased toward persons with more severe illness, detection may be less biased between hospitalized cases and deaths than that of outpatient influenza. A ratio of the number of influenza deaths to the number of influenza hospitalizations in a given population may provide some information on the relative severity of a pandemic influenza virus if a greater proportion of severe illness results in death than previously expected.

Limitations: The threshold for hospitalization can vary between populations and over time, depending on the capacity of the health care system. It will be important to better characterize this measure historically to establish an appropriate baseline and variability in the measure. As influenza activity increases, however, an increasing likelihood of death compared to all those with severe illness may be an important measure to understand as a possible indicator of strain on the capacity of the health system to provide supportive care.

3. Genetic markers of virulence

Strengths: Laboratory analysis will be available quickly upon detection of a novel virus. Genetic markers potentially associated with increased propensity for virulence may be identified from the results of this analysis.

Limitations: The association between these markers and severity in human populations is not well-understood. A clear understanding of the presence and absence of certain mutations and their corresponding population-level impact on the virus' pathogenicity in humans is lacking.

4. Animal immunopathologic experiments

Strengths: Soon after isolation of a novel influenza virus, experiments in ferrets can be performed to determine the clinical features of infection. Pathologic and immunologic studies of tissues and biological markers can indicate the extent of infection and the morbidity and mortality of infection relative to experimental infection with other seasonal, novel, and past pandemic viruses.

Limitations: Currently, this capability exists in only a few laboratories in the world which can implement animal studies in appropriate conditions. Results from ferret studies may not represent virulence in humans.

5. Percent of ED visits that resulted in hospitalization

Strengths: Data from electronic data sources may be the earliest source of clinical data available to characterize the spectrum of illness associated with pandemic influenza virus infection. Early analysis of basic data in an outbreak-affected area may provide a sense of the proportion of people presenting to medical care that require hospitalization or other supportive care.

Limitations: The threshold for hospitalization is known to vary from setting to setting, thus it will be important to have a data source that has a well-characterized baseline for comparison. Currently, there is limited access to historic data and therefore was not included in the current impact assessment. As these data sources are relatively new and expanding, additional analysis of such existing data may be needed for interpretation.

6. Percent of hospitalizations admitted to ICU

Strengths: If influenza testing is likely to be biased to persons with more severe illness, detection may be less biased between all hospitalized cases and those requiring ICU admission. The smaller and more well-defined population may allow for more complete ascertainment, and thus a more valid measure of whether a greater proportion of hospitalized cases require critical care than would be expected.

Limitations: The threshold for hospitalization and ICU care may vary between populations and over time, depending on the capacity of the health care system. Currently, there is limited access to historic data and therefore was not included in the current impact assessment. It will be important to better characterize this measure historically to establish an appropriate baseline and variability in the measure.

7. Rate of hospitalization

Strengths: Because influenza testing is more likely to be performed for serious illness, hospitalization for influenza-related causes is less likely to be under ascertained. This measure may provide an assessment of whether the burden of hospitalization is higher than expected and whether specific risk factors exist that increase the rate of hospitalization.

Limitations: The rate of hospitalization is a combination of both the attack rate and risk of hospitalization among ill persons. As a result, without a corresponding measure of attack rate, it is difficult to interpret whether an increased rate of hospitalization represents increased clinical severity of illness or greater incidence of illness in the population.

8. Excess deaths

Strengths: These data have been used for many decades to define a baseline and epidemic threshold for mortality due to pneumonia and influenza (P & I), and provide a well-characterized means to compare P & I mortality from year to year.

Limitations: The number of excess deaths observed in a population can be a misleading indicator of severity because it is a combination of both the attack rate and risk of death among ill persons. As a result, without a corresponding measure of attack rate, it is difficult to interpret whether an increased number of deaths represents increased clinical severity of illness or greater incidence of illness in the population.

C. Data quality evaluation

Because of the uncertainty of early findings, the assessment will continue to be reviewed and revised as the data warrant. Issues of data quality should factor into decisions about the inclusion of epidemiologic data in the impact assessment. The following are some data quality considerations:

- **Type of estimates available**: What is the source population? Who is excluded and are there any impacts caused by these exclusions? Do the data measure the factors meant to be measured?
- **Timeliness**: What is the time period for which the data were collected?
- **Geographic detail**: What is the geographical source of the data? What geographic regions do these data represent? If international, is the population, culture, and medical infrastructure similar to the United States?
- **Availability of historic information**: Do the current data have a historic record with which to compare and benchmark?
- **Statistical standards**: Are there any serious accuracy or methodological problems with the statistics?
- **Revisions to data**: Has the data been revised or corrected because of data quality or analysis issues?
- **Presentation of the information**: Are key materials to support correct interpretation, such as concepts, sources, and methods, provided? Are the data and results presented clearly?
- **Other cautions**: Is there any other relevant issue or caution that should be exercised in the use of the data?

Adapted from: The Australian Bureau of Statistics Data Quality Framework (1520.0)

D. Data on measures of transmission and severity used to scale examples of past seasons and pandemics

Table D.1: Measures from 1918 pandemic, all age groups.

**TRANSMISSION**		**Range of observed values**			
**Parameter**		**Low**	**High**	**Score**	**Reference**
**1**	Cumulative incidence of ILI, community	8.8	39.1	5	(7)
**2**	R_o: Basic Reproductive Number	--	2	5	(8)
**SEVERITY**		**Range of observed values**			
**Parameter**		**Low**	**High**	**Score**	**Reference**
**1**	Symptomatic case-fatality ratio	--	2.04%	7	(*9*)

Table D.2: Measures from 1957 pandemic, all age groups.

**TRANSMISSION**		**Range of observed values**			
**Parameter**		**Low**	**High**	**Score**	**Reference**
**1**	Cumulative incidence of ILI, community	20%	48%	5	(*10, 11*)
**2**	Cumulative incidence of laboratory-confirmed illness	18.5%	56.8%	4	(*12, 13*)
**3**	Household secondary attack rate	8.4%	23.0%	4	(*10*)
**4**	R_o: Basic Reproductive Number	1.68	1.68	4	(*14*)
**SEVERITY**		**Range of observed values**			
**Parameter**		**Low**	**High**	**Score**	**Reference**
**1**	Symptomatic case-fatality ratio	0.1%	0.3%	4	(*15*)

Table D.3: Measures from 1968 pandemic, all age groups

TRANSMISSION		Range of observed values			
**Parameter**		**Low**	**High**	**Score**	**Reference**
**1**	Cumulative incidence of illness, non-confirmed	15%	43%	4	(*16-20*)
**2**	Cumulative incidence of illness, confirmed	10.4%	32%	4	(*16-18, 21*)
**3**	Cumulative incidence of infection, confirmed	15%	15%	2	(*22*)
**4**	Household secondary attack rate, non-confirmed	20%	20%	4	(*18*)
**5**	R_o: Basic Reproductive Number	1.06	2.01	4	(*23*)
**SEVERITY**		**Range of observed values**			
**Parameter**		**Low**	**High**	**Score**	**Reference**
**1**	Symptomatic case-fatality ratio	--	0.05%	3	(*24*)

Table D.4: Measures from 2009 pandemic, all age groups

TRANSMISSION		Range of observed values			
**Parameter**		**Low**	**High**	**Score**	**Reference**
**1**	Cumulative incidence of symptomatic illness, community, confirmed	--	19.9	3	(*25*)
**2**	Cumulative incidence of symptomatic illness, workplace, non-confirmed	--	17.5	3	(*26*)
**3**	Household symptomatic secondary attack rate, non-confirmed	11	24	3	(*3, 27-29*)
**4**	Household symptomatic secondary attack rate, confirmed	4	6	1	(*27, 28*)
**5**	Peak of ILI activity, percent of clinic visits	--	7.7	3	(*30*)
**6**	R_o: Basic Reproductive Number	1.0	3.3	3	(*31*)
**SEVERITY**		**Range of observed values**			
**Parameter**		**Low**	**High**	**Score**	**Reference**
**1**	Case-fatality ratio	0.007%	0.048%	2	(*5, 6*)
**2**	Case-hospitalization ratio	0.16%	1.44%	2	(*5, 6*)
**3**	Ratio, deaths:hospitalizations	1.8%	8%	2	(*6, 25, 32, 33*)

Table D.5: Measures from 1977-78 season, all age groups

TRANSMISSION		Range of observed values			
**Parameter**		**Low**	**High**	**Score**	**Reference**
**1**	Cumulative incidence of ILI, community, non-confirmed	--	48%	3	(*34*)
**2**	Cumulative incidence of symptomatic illness, community, confirmed	1.0%	20.1%	2	(*35, 36*)
**3**	Cumulative incidence of infection, community, confirmed	2.2%	31%	2	(*37, 38*)
**4**	Household secondary attack rate, infection	--	16%	2	(*37*)
**SEVERITY**		**Range of observed values**			
**Parameter**		**Low**	**High**	**Score**	**Reference**
**1**	Influenza excess mortality*	2.2 per 100,000	12.7 per 100,000	2	(*39-41*)
**2**	Case-hospitalization ratio	0.55%	1.60%	2	(*42, 43*)
**3**	Ratio, deaths:hospitalization	--	4.9%	2	(*43*)

*These measures to not directly estimate the clinical severity, thus the score for these measures was estimated accounting for the corresponding level of transmissibility.

Table D.6: Measures from 2006-07 season, all age groups

TRANSMISSION		Range of observed values			
**Parameter**		**Low**	**High**	**Score**	**Reference**
**1**	Peak of ILI activity, percent of clinic visits	--	3.5%	1	(*44*)
**SEVERITY**		**Range of observed values**			
**Parameter**		**Low**	**High**	**Score**	**Reference**
**1**	Influenza excess mortality*	--	1.55 per 100,000	2	(*39*)
**2**	Influenza excess hospitalization*	--	26.1 per 100,000	1	(*45*)
**3**	Ratio, deaths:hospitalizations	--	5.9%	2	(*45, 46*)

*These measures to not directly estimate the clinical severity, thus the score for these measures was estimated accounting for the corresponding level of transmissibility.

Table D.7: Measures from 2007-08 season, all age groups

**TRANSMISSION**		**Range of observed values**			
**Parameter**		**Low**	**High**	**Score**	**Reference**
**1**	Cumulative incidence of symptomatic illness, community, confirmed	3.1	10.8	2	(*47*)
**2**	Peak of ILI activity, percent of clinic visits	--	6%	2	(*48*)
**SEVERITY**		**Range of observed values**			
**Parameter**		**Low**	**High**	**Score**	**Reference**
**1**	Influenza excess mortality*	--	3.91 per 100,000	3	(*46*)
**2**	Influenza excess hospitalization*	--	66.8 per 100,000	3	(*45*)
**4**	Ratio, deaths:hospitalizations	--	5.9%	2	(*45, 46*)

*These measures to not directly estimate the clinical severity, thus the score for these measures was estimated accounting for the corresponding level of transmissibility.

E. References:

1. **Zachary, I.G. & Johnson, K.M.** Hong Kong influenza in the Panama Canal Zone. First epidemic by a new variant in the Western Hemisphere. *Am J Trop Med Hyg. 18* (6): 1048-56 (1969).

2. **Becker, N.G. et al.** Type and quantity of data needed for an early estimate of transmissibility when an infectious disease emerges. *Euro Surveill. 15* (26) (2010).

3. **Cauchemez, S. et al.** Household transmission of 2009 pandemic influenza A (H1N1) virus in the United States. *N Engl J Med. 361* (27): 2619-27 (2009).

4. **Garske, T. et al.** Assessing the severity of the novel influenza A/H1N1 pandemic. *BMJ. 339*: b2840 (2009).

5. **Presanis, A.M. et al.** The severity of pandemic H1N1 influenza in the United States, from April to July 2009: a Bayesian analysis. *PLoS Med. 6* (12): e1000207 (2009).

6. **Reed, C. et al.** Estimates of the prevalence of pandemic (H1N1) 2009, United States, April-July 2009. *Emerg Infect Dis. 15* (12): 2004-7 (2009).

7. **Frost, W.H.** Statistics of Influenza Morbidity: with special reference to certain factors in case incidence and case fatality *Public Health Records. 36*: 13 (1920).

8. **Mills, C.E. et al.** Transmissibility of 1918 pandemic influenza. *Nature. 432* (7019): 904-6 (2004).

9. **Collins, S.D.** Age and Sex Incidence of Influenza and Pneumonia Morbidity and Mortality in the Epidemic of 1928-29. *Public Health Records. 46* (33): 1909-1937 (1931).

10. **Chin, T.D. et al.** Morbidity and mortality characteristics of Asian strain influenza. *Public Health Rep. 75* (2): 149-158 (1960).

11. **Dunn, F.L. et al.** Epidemiologic studies of Asian influenza in a Louisiana parish. *Am J Hyg. 70*: 351-71 (1959).

12. **Jordan, W.S., Jr. et al.** A study of illness in a group of Cleveland families. XVII. The occurrence of Asian influenza. *Am J Hyg. 68* (2): 190-212 (1958).

13. **Carey, D.E. et al.** Community-wide epidemic of Asian strain influenza; clinical and subclinical illnesses among school children. *J Am Med Assoc. 167* (12): 1459-63 (1958).

14. **Longini, I.M., Jr. et al.** Containing pandemic influenza with antiviral agents. *Am J Epidemiol. 159* (7): 623-33 (2004).

15. **Payne, A.M.** Some aspects of the epidemiology of the 1957 influenza pandemic. *Proc R Soc Med. 51* (12): 1009-15 (1958).

16. **Mogabgab, W.J. & Leiderman, E.** Immunogenicity of 1967 polyvalent and 1968 Hong Kong influenza vaccines. *JAMA. 211* (10): 1672-6 (1970).

17. **Waldman, R.H. & Coggins, W.J.** Influenza immunization: field trial on a university campus. *J Infect Dis. 126* (3): 242-8 (1972).

18. **Davis, L.E. et al.** Hong Kong influenza: the epidemiologic features of a high school family study analyzed and compared with a similar study during the 1957 Asian influenza epidemic. *Am J Epidemiol. 92* (4): 240-7 (1970).

19. **Piraino, F.F. et al.** Outbreak of Hong Kong influenza in Milwaukee, winter of 1968-69. *Public Health Rep. 85* (2): 140-50 (1970).

20. **Sharrar, R.G.** National influenza experience in the USA, 1968-69. *Bull World Health Organ. 41* (3): 361-6 (1969).

21. **Waldman, R.H. et al.** An evaluation of influenza immunization: influence of route of administration and vaccine strain. *Bull World Health Organ. 41* (3): 543-8 (1969).

22. **Foy, H.M. et al.** Longitudinal studies of types A and B influenza among Seattle schoolchildren and families, 1968-74. *J Infect Dis. 134* (4): 362-9 (1976).

23. **Jackson, C. et al.** Estimates of the transmissibility of the 1968 (Hong Kong) influenza pandemic: evidence of increased transmissibility between successive waves. *Am J Epidemiol. 171* (4): 465-78 (2010).

24. **CDC.** Interim Pre-pandemic Planning Guidance: Community Strategy for Pandemic Influenza Mitigation in the United States. CS108488 (2007).

25. **Shrestha, S.S. et al.** Estimating the burden of 2009 pandemic influenza A (H1N1) in the United States (April 2009-April 2010). *Clin Infect Dis. 52 Suppl 1*: S75-82 (2011).

26. **Gindler, J. et al.** A model survey for assessing 2009 pandemic influenza A (H1N1) virus disease burden in the workplace. *Clin Infect Dis. 52 Suppl 1*: S173-6 (2011).

27. **Jhung, M.A. et al.** Epidemiology of 2009 pandemic influenza A (H1N1) in the United States. *Clin Infect Dis. 52 Suppl 1*: S13-26 (2011).

28. **Morgan, O.W. et al.** Household transmission of pandemic (H1N1) 2009, San Antonio, Texas, USA, April-May 2009. *Emerg Infect Dis. 16* (4): 631-7 (2010).

29. **France, A.M. et al.** Household transmission of 2009 influenza A (H1N1) virus after a school-based outbreak in New York City, April-May 2009. *J Infect Dis. 201* (7): 984-92 (2010).

30. **Brammer, L. et al.** Surveillance for influenza during the 2009 influenza A (H1N1) pandemic-United States, April 2009-March 2010. *Clin Infect Dis. 52 Suppl 1*: S27-35 (2011).

31. **Boelle, P.Y. et al.** Transmission parameters of the A/H1N1 (2009) influenza virus pandemic: a review. *Influenza Other Respi Viruses. 5* (5): 306-16 (2011).

32. **Skarbinski, J. et al.** Hospitalized patients with 2009 pandemic influenza A (H1N1) virus infection in the United States--September-October 2009. *Clin Infect Dis. 52 Suppl 1*: S50-9 (2011).

33. **Jhung, M.A. et al.** Preliminary results of 2009 pandemic influenza surveillance in the United States using the Aggregate Hospitalization and Death Reporting Activity. *Influenza Other Respi Viruses. 5* (5): 321-7 (2011).

34. **Sobal, J. & Loveland, F.C.** Infectious disease in a total institution: a study of the influenza epidemic of 1978 on a college campus. *Public Health Rep. 97* (1): 66-72 (1982).

35. **Monto, A.S. et al.** Prevention of Russian influenza by amantadine. *JAMA. 241* (10): 1003-7 (1979).

36. **Wright, P.F. et al.** Differing virulence of H1N1 and H3N2 influenza strains. *Am J Epidemiol. 112* (6): 814-9 (1980).

37. **Fox, J.P. et al.** Influenzavirus infections in Seattle families, 1975-1979. I. Study design, methods and the occurrence of infections by time and age. *Am J Epidemiol. 116* (2): 212-27 (1982).

38. **Monto, A.S. et al.** Tecumseh study of illness. XIII. Influenza infection and disease, 1976-1981. *Am J Epidemiol. 121* (6): 811-22 (1985).

39. Estimates of deaths associated with seasonal influenza --- United States, 1976-2007. *MMWR Morb Mortal Wkly Rep. 59* (33): 1057-62 (2010).

40. **Thompson, W.W. et al.** Mortality associated with influenza and respiratory syncytial virus in the United States. *JAMA. 289* (2): 179-86 (2003).

41. **Thompson, W.W. et al.** Estimates of US influenza-associated deaths made using four different methods. *Influenza Other Respi Viruses. 3* (1): 37-49 (2009).

42. **Barker, W.H. & Mullooly, J.P.** Impact of epidemic type A influenza in a defined adult population. *Am J Epidemiol. 112* (6): 798-811 (1980).

43. **Simonsen, L. et al.** The impact of influenza epidemics on mortality: introducing a severity index. *Am J Public Health. 87* (12): 1944-50 (1997).

44. Update: Influenza activity--United States and worldwide, 2006-07 season, and composition of the 2007-08 influenza vaccine. *MMWR Morb Mortal Wkly Rep. 56* (31): 789-94 (2007).

45. **Zhou, H. et al.** Hospitalizations Associated with Influenza and Respiratory Syncytial Virus (RSV) in the US from the 1993-94 through 2005-06 Seasons. *Options for the Control of Influenza VII*. Hong Kong, 2010.

46. **Cheng, P.Y. et al.** Timely Estimates of Seasonal Influenza-Associated Deaths in the United States Using CDC 122 Cities Mortality Reporting System Data. *Options for the Control of Influenza VII*. Hong Kong, 2010.

47. **Monto, A.S. et al.** Comparative efficacy of inactivated and live attenuated influenza vaccines. *N Engl J Med. 361* (13): 1260-7 (2009).

48. Influenza activity--United States and worldwide, 2007-08 season. *MMWR Morb Mortal Wkly Rep. 57* (25): 692-7 (2008).
